# Draft Genome Sequence of Uncultured Upland Soil Cluster *Gammaproteobacteria* Gives Molecular Insights into High-Affinity Methanotrophy

**DOI:** 10.1128/genomeA.00047-17

**Published:** 2017-04-27

**Authors:** Collin R. Edwards, Tullis C. Onstott, Jennifer M. Miller, Jessica B. Wiggins, Wei Wang, Charles K. Lee, S. Craig Cary, Stephen B. Pointing, Maggie C. Y. Lau

**Affiliations:** a Department of Geosciences, Princeton University, Princeton, New Jersey, USA; b Genomics Core Facility, Lewis-Sigler Institute for Integrative Genomics, Princeton University, Princeton, New Jersey, USA; c School of Science, University of Waikato, Hamilton, New Zealand; d International Centre for Terrestrial Antarctic Research (ICTAR), University of Waikato, Hamilton, New Zealand; e Institute for Applied Ecology New Zealand, School of Science, Auckland University of Technology, Auckland, New Zealand

## Abstract

Aerated soils form the second largest sink for atmospheric CH_4_. A near-complete genome of uncultured upland soil cluster *Gammaproteobacteria* that oxidize CH_4_ at <2.5 ppmv was obtained from incubated Antarctic mineral cryosols. This first genome of high-affinity methanotrophs can help resolve the mysteries about their phylogenetic affiliation and metabolic potential.

## GENOME ANNOUNCEMENT

Aerated soils remove 4 to 6% of CH_4_ from the atmosphere (1) as a result of high-affinity methanotrophy that has been demonstrated by the detection of the high-affinity form of particulate methane monooxygenase, encoded by *pmo* genes (2–6). In the absence of isolates and genomes, phylogenetic identity and physiology of atmospheric methane-oxidizing bacteria have remained a mystery (7). Here, we report a near-complete genome of uncultured upland soil cluster *Gammaproteobacteria* (USC**γ**) obtained from incubated mineral cryosols (pH 8.51) collected from Taylor Dry Valley, Antarctica. These samples exhibited CH_4_ oxidation when incubated with 2.5 ppmv at 4°C and 10°C, and were found to contain USC**γ**-like *pmo*A genes (8).

DNA was extracted using PowerSoil total DNA/RNA isolation kit (MO BIO Laboratories, Inc., CA), purified and concentrated using Microcon centrifugal filters 100 (EMD Millipore Corp., MA). Four metagenomic libraries were prepared using the PrepX DNA library kit and the automated Apollo 324^TM^ robotic system (WaferGen Bio-systems, Inc., CA), pooled at equal molar amount and sequenced on Illumina HiSeq 2500 (Illumina Inc., CA). Paired-end reads (2 × 100 nucleotides [nt]; mean insert size of 350 bp) were quality filtered (9). High-quality reads (average 75.7 ± 7.5 million reads per sample) were coassembled using IDBA_UD v1.1.1 (default settings with precorrection) (10). Protein-encoding genes were predicted using Prodigal v2.6.1 (11) and searched for *pmo*A genes. All scaffolds were subjected to binning by an interactive approach (12) and MetaBAT (13).

A high-quality genome bin containing a *pmo*A gene 100% identical to one of our USC**γ**-like *pmo*A clone sequences was identified. Reads mapped to the scaffolds in this genome bin were reassembled using IDBA_UD (default settings). CheckM (14) determined that it was 89.99 to 91.9% complete with 0 to 0.09% contamination and 0% strain heterogeneity. Annotation was performed using Prokka v1.12-beta (15), BLAST (16), and KEGG Automatic Annotation Server v2.1 (17). A single copy of twenty 30S and thirty-one 50S ribosomal proteins, one 5S rRNA operon, a single copy of 16S and 23S rRNA gene fragments, 38 tRNA genes, and 3,012 coding sequences were retrieved. The 488 nt-long 16S rRNA gene shared 99% identity to uncultured bacteria (JQ684308, HM445440, and DQ823229) and 94% identity to *Thioalkalivibrio* (NR_074692) and *Ectothiorhodospira* (NR_125567) of *Chromatiales*, which is phylogenetically closely related to *Methylococcales* but includes no methanotrophs.

The USCγ draft genome contained the complete operon of *pmo*CAB. The *pmo*A gene shared 72% identity with *Methylocaldum szegediense* (*Methylococcales*). Genes encoding methanol dehydrogenase and accessory proteins, enzymes for the tetrahydrofolate and tetrahydromethanopterin-linked C1 transfer pathways, and two formate dehydrogenases were identified. Although gene encoding formaldehyde dehydrogenase (FALDH) was missing, genes for pyrroloquinoline quinone (PQQ) synthesis were identified, indicative of the potential use of PQQ-dependent FALDH. As with the genomes of other gammaproteobacterial methanotrophs, the USC**γ** draft genome encodes all essential genes for a complete serine biosynthesis pathway for formaldehyde assimilation. Genes encoding enzymes for nitrogen metabolism were also identified. Manual annotation and phylogenetic and comparative analyses are in progress to pinpoint the phylogenetic affiliation of USCγ and to elucidate differences in the metabolic potential between high- and low-affinity methanotrophs.

### Accession number(s).

This whole-genome shotgun project and the USCγ draft genome have been deposited at DDBJ/ENA/GenBank under the accession no. MUGK00000000. The version described in this paper is version MUGK01000000.
