# Erratum for Edwards et al., “Draft Genome Sequence of Uncultured Upland Soil Cluster *Gammaproteobacteria* Gives Molecular Insights into High-Affinity Methanotrophy”

**DOI:** 10.1128/genomeA.00962-17

**Published:** 2017-09-07

**Authors:** Collin R. Edwards, Tullis C. Onstott, Jennifer M. Miller, Jessica B. Wiggins, Wei Wang, Charles K. Lee, S. Craig Cary, Stephen B. Pointing, Maggie C. Y. Lau

**Affiliations:** a Department of Geosciences, Princeton University, Princeton, New Jersey, USA; b Genomics Core Facility, Lewis-Sigler Institute for Integrative Genomics, Princeton University, Princeton, New Jersey, USA; c School of Science, University of Waikato, Hamilton, New Zealand; d International Centre for Terrestrial Antarctic Research (ICTAR), University of Waikato, Hamilton, New Zealand; e Institute for Applied Ecology New Zealand, School of Science, Auckland University of Technology, Auckland, New Zealand

## ERRATUM

Volume 5, no. 17, e00047-17, 2017, https://doi.org/10.1128/genomeA.00047-17. Page 2: Lines 11–14 should read as follows: “…Unlike the genomes of other gammaproteobacterial methanotrophs, the USCγ draft genome contains nearly all essential genes for a complete serine biosynthesis pathway for formaldehyde assimilation. However, the enzymes unique to the typical RUMP pathway, hexulose-6-phosphate synthase and hexulose phosphate isomerase, were not detected….”

